# Analyzing the impact of *Mycobacterium tuberculosis* infection on primary human macrophages by combined exploratory and targeted metabolomics

**DOI:** 10.1038/s41598-020-62911-1

**Published:** 2020-04-27

**Authors:** Frank Vrieling, Sarantos Kostidis, Herman P. Spaink, Mariëlle C. Haks, Oleg A. Mayboroda, Tom H. M. Ottenhoff, Simone A. Joosten

**Affiliations:** 1 Department of Infectious Diseases, Leiden University Medical Center, Leiden, The Netherlands; 2 Center for Proteomics and Metabolomics, Leiden University Medical Center, Leiden, The Netherlands; 3 Institute of Biology, Leiden University, Leiden, The Netherlands

**Keywords:** Preclinical research, Infection, Inflammation, Innate immune cells, Pathogens, Infection, Inflammation

## Abstract

The pathogenic success of *Mycobacterium tuberculosis* (*Mtb*) is tightly linked to its ability to recalibrate host metabolic processes in infected host macrophages. Since changes in cellular metabolic intermediates or pathways also affect macrophage function in response to pathogens, we sought to analyse specific metabolic alterations induced by *Mtb* infection. Stimulation of macrophages with *Mtb* lysate or lipopolysaccharide (LPS) induced a relative increase in glycolysis versus oxidative phosphorylation. Cellular metabolomics revealed that *Mtb* infection induced a distinct metabolic profile compared to LPS in both M1 and M2 macrophages. Specifically, *Mtb* infection resulted in elevated intracellular levels of nicotinamide adenine dinucleotide (NAD^+^), creatine, creatine phosphate and glutathione compared to uninfected control macrophages. Correspondingly, RNA-sequencing datasets showed altered gene expression of key metabolic enzymes involved in NAD^+^, creatine, glucose and glutamine metabolism (*e.g NAMPT, SLC6A8, HK2*) in *Mtb*-infected M2 macrophages. These findings demonstrate clear modulation of host macrophage metabolic pathways by *Mtb* infection.

## Introduction

*Mycobacterium tuberculosis* (*Mtb*) is the causative pathogen of tuberculosis (TB) and responsible for over a million deaths annually^1^. *Mtb* is transmitted through inhalation of aerosol particles and transported to the lungs, where it infects alveolar macrophages and avoids eradication through interfering with innate antimicrobial mechanisms. Infected cells are sequestered at the core of the TB granuloma as part of the host immune response, a confined niche where *Mtb* can reside in a dormant state for decades before potential disease reactivation^2,3^. However, in order to persist *Mtb* must overcome the limitations set by the anti-mycobacterial microenvironment of the granuloma, which include hypoxia^4^ and nutrient scarcity^5^. These conditions compel *Mtb* to switch from using carbohydrates to lipids and cholesterol as primary carbon source during later stages of infection, as part of its transition to a dormant state^6–9^. Several studies have demonstrated that *Mtb* is able to reprogram macrophage metabolism, and these adaptations are thought to be essential for its pathogenic success^10–12^.

Besides providing the necessary nutrients, metabolic changes induced by *Mtb* could also rewire the activation state and anti-microbial effector functions of infected macrophages. Over recent years many studies in the emerging field of immunometabolism have attempted to define the associations between macrophage metabolic states and their immunological responses^13^. The outcome of macrophage immunometabolism is largely determined by the balance between glycolysis and mitochondrial metabolism through oxidative phosphorylation (OXPHOS) of tricarboxylic acid cycle (TCA) intermediates^14,15^. Glycolysis is associated with classical pro-inflammatory macrophages activated with IFNγ and/or the Toll-like receptor (TLR) 4 ligand lipopolysaccharide (LPS)^16^, and OXPHOS with the alternatively activated anti-inflammatory phenotype induced by the T_H_2 cytokines interleukin-(IL)-4 and IL-13^17^. Activation of myeloid cells and T cells has been demonstrated to enhance aerobic glycolysis^18,19^, resembling a process first observed in cancer cells by Otto Warburg and therefore known as the Warburg effect^20^. The Warburg effect supports pro-inflammatory effector functions through rapid production of ATP and other necessary metabolic intermediates. Several studies reported increased lactate production or glycolytic enzyme expression in human and murine macrophages or lung tissue after *Mtb* infection^21–24^, implying that glycolysis is induced as part of the host anti-mycobacterial response. However, stimulation with different pathogens or TLR ligands has since been shown to lead to more complex metabolic phenotypes in myeloid cells than what simply can be explained by the Warburg effect, including changes in lipid, cholesterol and amino acid metabolism^25^. Importantly, *Mtb* and other mycobacteria have been shown to manipulate macrophage lipid metabolism, leading to the formation of lipid-loaded foam cells which constitute a preferred niche for mycobacterial persistence^8,11,26–28^.

Considering the importance of metabolic adaptations for *Mtb* killing and survival, several studies aimed to dissect the precise impact of the bacterium on macrophage metabolism by cellular metabolomics^29–31^. However, these relied on phorbol 12-myristate 13-acetate (PMA)-activated macrophage-like THP-1 cells as a model for macrophage infection, which significantly differ from primary macrophages in terms of polarization and response to stimuli^32,33^. To address this critical gap in knowledge, we have here studied the effect of *Mtb* infection on primary human macrophage metabolism using not only untargeted liquid chromatography-mass spectrometry (LC-MS) metabolomics but also targeted ^1^H-nuclear magnetic resonance (NMR) spectroscopy^34^.

## Results

### *Mtb* lysate and LPS induced glycolytic metabolism in human macrophages

**Figure 1:**
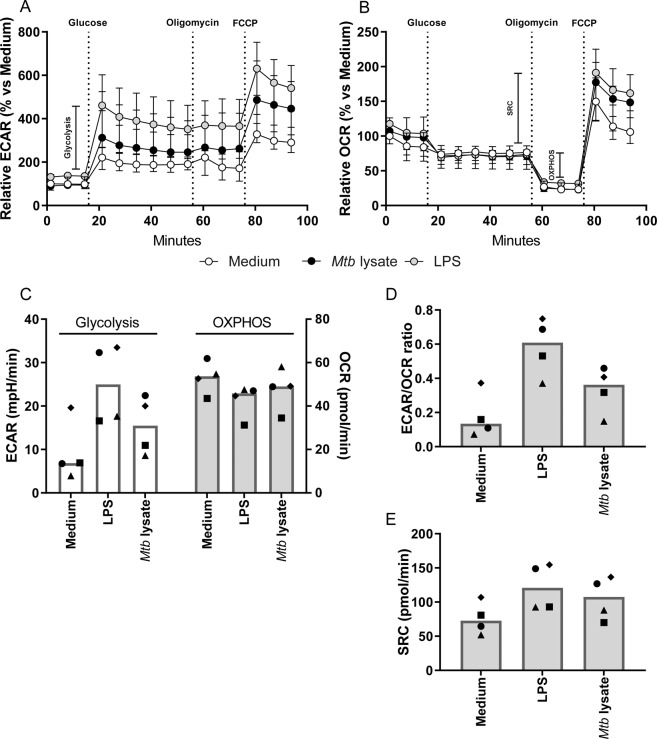
Stimulation with LPS or *Mtb* lysate induced a glycolytic shift in primary human macrophages. M2 macrophages were stimulated with medium (white circles), *Mtb* lysate (10 µg/ml; black circles) or LPS (100 ng/ml; grey circles) for 24 h. (**A**) Macrophage extracellular acidification rate (ECAR) and (**B**) oxygen-consumption rate (OCR) were measured during sequential injections of D-glucose (10 mM), oligomycin (1 µM) and FCCP (2 µM). (**C**) Glycolysis (white bars) as determined by the difference in ECAR pre- and post-glucose injection, and OXPHOS (grey bars) as the difference in OCR pre- and post-oligomycin injection. (**D**) ECAR/OCR ratio. (**E**) Spare respiratory capacity as determined by the difference in OCR pre-oligomycin and post-FCCP injection. Each symbol represents an individual donor, and bars represent group medians (n = 4). Data is depicted as medians with ranges (n = 4).

*In vitro* stimulation with TLR ligands or whole pathogen lysates is commonly used to model immune cell activation in response to bacterial infection, and has previously been demonstrated to modulate myeloid cell metabolism^18,25^. To validate whether primary human macrophage metabolism was truly affected by *Mtb* stimulation, macrophage colony-stimulating factor (M-CSF)-derived primary human macrophages (M2) were stimulated with *Mtb* lysate (10 µg/ml) as a model for *Mtb* infection and their metabolic activity was analyzed using a Seahorse XF Analyzer. LPS (100 ng/ml), a TLR4 ligand which is known to induce glycolysis in macrophages, and culture medium were used as a positive and negative control for metabolic skewing, respectively. Cellular glycolysis (Fig. 1A), OXPHOS and spare respiratory capacity (SRC) (Fig. 1B) were determined after a series of injections with D-glucose, ATP synthase inhibitor oligomycin and mitochondrial uncoupling agent FCCP. As expected, LPS stimulation showed a trend towards increased glycolysis-related acidification, while simultaneously decreasing macrophage mitochondrial respiration compared to medium control (Fig. 1C,D), albeit with a greater SRC (Fig. 1E). *Mtb* lysate induced similar tendencies for both extracellular acidification rate (ECAR)/oxygen consumption rate (OCR) ratio (Fig. 1C,D) and SRC (Fig. 1E), although the magnitude of this effect was less pronounced compared to LPS. Taken together, both stimulation with *Mtb* and LPS seem to result in metabolic skewing towards increased glycolysis while simultaneously decreasing OXPHOS in primary human macrophages.

### Exploratory metabolomics of *Mtb*-infected macrophages

Since mycobacterial products are able to redirect macrophage metabolism, we sought to further characterize the effects of live *Mtb* infection on macrophage metabolism using exploratory metabolomics. Therefore, we generated a set consisting of granulocyte-macrophage colony-stimulating factor (GM-CSF) (M1) and M-CSF (M2) differentiated macrophages from six healthy blood bank donors which were either infected with *Mtb*-H37Rv, stimulated with LPS (100 ng/ml) or left untreated and subsequently harvested at either 4 or 24 h post-infection for metabolite extraction. Supplementary Fig. 1 (Fig. S1A–D) shows an exploratory analysis of the resulting dataset (peak intensities of 270 masses) by Principal Component Analysis (PCA); the resulting score plot is colored according to main possible sources of variance in the LC-MS data. A dichotomy in cell type indicating metabolic differences between M1 and M2 macrophages was observed in the first two principal components, explaining 24% of total variance (Fig. S1B). However, high inter-individual heterogeneity also strongly contributed to the variance explained by the first two components. Therefore, a multilevel PCA model was fitted on the dataset to separate the between-donor and within-donor data variation^35^ (Fig. S1E–G). This model successfully reduced the donor related variability (Fig. S1E), while retaining the metabolic effects of cell type (Fig. S1F) within the first two components, explaining 19% of total variance. To compensate for this difference in metabolic profile at baseline between M1 and M2 macrophages, distinct multilevel PCA models were fitted for both cell types (Fig. S2) to visualize potential effects of *Mtb* infection or LPS stimulation. The resulting score plots showed improved separation based on infection/treatment status in M2 macrophages (Fig. S2F), however this was not clearly observable in M1 macrophages (Fig. S2C).

**Figure 2:**
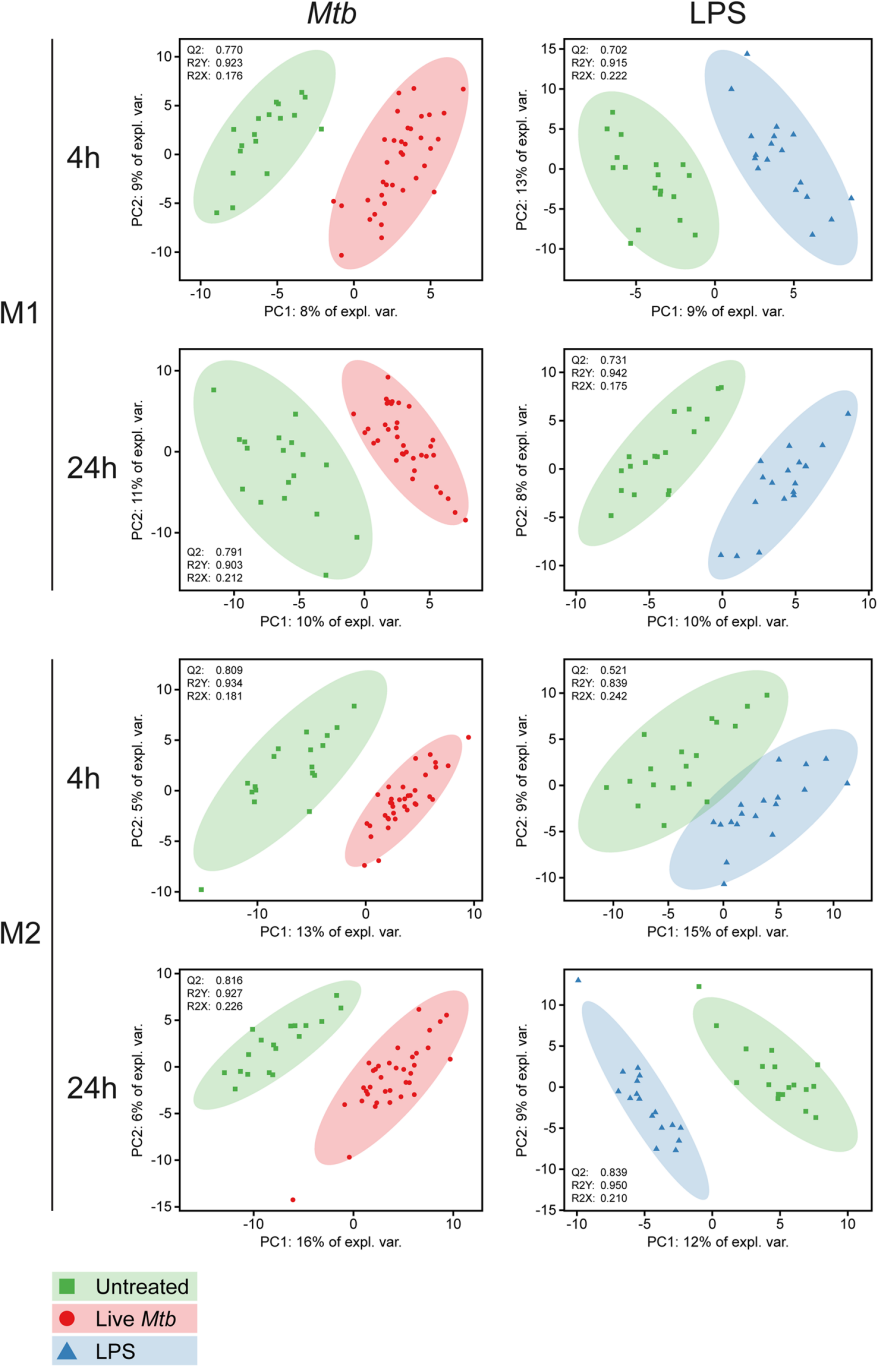
PLS-DA models of M1 and M2 macrophages after 4 h or 24 h of *Mtb* infection or LPS stimulation. M1 and M2 macrophages were either infected with *Mtb* at a MOI of 10:1, stimulated with LPS (100 ng/ml) or left untreated. Cells were lysed at 4 h and 24 h post-infection/stimulation and intracellular metabolites were subsequently extracted and measured by LC-MS. Multilevel PLS-DA models were fitted for each group/time point comparison in M1 and M2 macrophages and score plots of the resulting eight models with associated quality metrics (Q2/R2Y/R2X) are displayed. Each point represents one technical replicate derived from six biological blood bank donors. Untreated samples are depicted as green squares, LPS stimulated samples as blue triangles and *Mtb* infected samples as red dots.

For a more focused analysis of the effect of *Mtb* infection or LPS stimulation on macrophage metabolism, we performed group separation at specific time points per stimulation using Partial Least Squares Discrimination Analysis (PLS-DA) and extracted Variable Importance in Projection (VIP) scores from the resulting models to identify which masses carried the highest classification weight for each individual comparison. Separate multilevel PLS-DA models were built for each combination of cell type and time point using treatment group status as class variable. Resulting score plots and cross-validated model quality characteristics are displayed in Fig. 2. All models showed good predictive capabilities as evidenced by high Q2 and R2Y scores (>0.5 and >0.8 respectively), signifying clear metabolic effects of both *Mtb* infection and LPS stimulation for each cell type and time point compared to untreated control. Next, VIP scores were extracted from the first component of each PLS-DA model to examine which measured variables explained the largest proportion of data variance in each model. Volcano plots of metabolite VIP scores versus their respective regression coefficients for each individual PLS-DA model are displayed in Supplementary Fig. S3. In total, 46 masses reached a combined high VIP score of ≥2 and associated regression coefficient of ≥0.1 or ≤−0.1 in at least one model.

**Figure 3:**
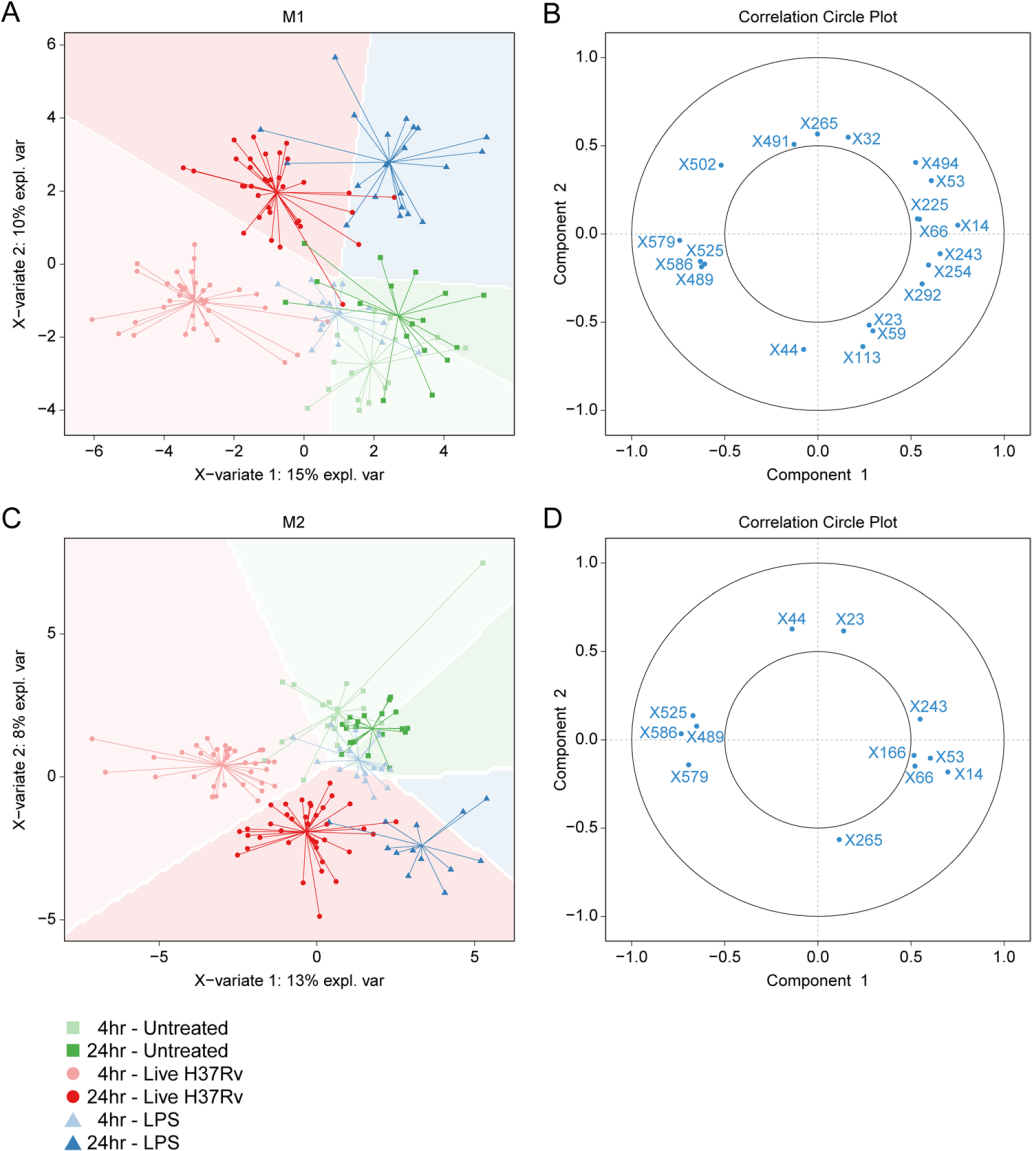
Group separation by selected metabolites with high Variable Importance for Projection (VIP) scores. Multilevel PLS-DA models were fitted on all samples using a selection of 46 metabolites which reached VIP scores of ≥2 and associated regression coefficients of ≤−0.1 or ≥0.1 in any pairwise PLS-DA model described in Fig. 2. Score plots of the resulting model for M1 (**A**) and M2 (**C**) macrophages (M2) are displayed. Untreated samples are depicted as green squares, LPS stimulated samples as blue triangles and *Mtb* infected samples as red dots. Shade of symbol color reflects time point (4 h = light, 24 h = dark). Strongly correlated metabolites (threshold: ≥0.5) are displayed in circle plots for both the M1 (**B**) and the M2 (**C**) PLS-DA model.

To verify the discriminatory capacity of this selection of masses, separate PLS-DA models were fitted on all combined samples derived from either M1 or M2 macrophages using only these 46 variables (Fig. 3A,C). Both cell type models still showed good class separation of *Mtb*-infected cells at 4 and 24 h and LPS-treated cells at 24 h compared to untreated controls samples based on the first two components. Circle correlation plots visualizing the correlation between individual variables and the first two components revealed 20 and 12 metabolites in M1 (Fig. 3B) and M2 (Fig. 3D) macrophages, respectively, with relatively good correlation scores (≥0.5), which combined constituted a total of 21 unique masses. Tentative annotations of these masses with degree of certainty are shown in Table 1, and boxplots of metabolite peak areas are displayed in Supplementary Fig. S4. In both cell types, LPS treatment was associated with increased levels of masses annotated as adenosine (X265, m/z = 269.104), nicotinamide (X32, m/z = 123.055) and propionylcarnitine (X166, m/z = 218.138), while *Mtb* infection showed high correlation with relatively large masses (X489, m/z = 382.189; X579, m/z = 458.249; X586, m/z = 470.241) which could not be annotated based on database searches. These unknown structures are fragments of either proteins or lipids for which the number of possible assignments cannot be reduced to a minimum required for tentative annotation.

**Table 1:** Tentative annotation of selected masses.

Nr.	m/z	ID	Adduct	Error (ppm)	ID level
X14	104.107	Choline	H^+^	6	3
X23	116.071	Proline	H^+^	1	2
X32	123.055	Nicotinamide	H^+^	1	2
X44	133.061	L-Asparagine	H^+^	2	2
X53	142.026	Dimethylglycine	K^+^	3	2
X59	146.996	NA	—	—	4
X66	149.063	Glutamate	H^+^	1	2
X113	184.073	3-Dehydroxycarnitine	K^+^	1	3
X166	218.138	Propionylcarnitine	H^+^	1	3
X225	249.045	Unknown peptide	H^+^	—	4
X243	258.110	Glycerophosphocholine	H^+^	1	3
X254	263.086	Hydroxysebacate	2Na-H	3	3
X265	268.104	Adenosine	H^+^	1	3
X292	280.092	Glycerophosphocholine	Na^+^	1	3
X489	382.189	Unknown peptide	—	—	4
X491	383.101	NA	—	—	—
X494	385.175	NA	—	—	—
X502	388.269	NA	—	—	—
X525	415.224	NA	—	—	—
X579	459.249	NA	—	—	—
X586	470.241	NA	—	—	—

### Targeted metabolomics of *Mtb*-infected macrophages by ^1^H-NMR spectroscopy

While the LC-MS approach clearly demonstrated that *Mtb* infection greatly impacts the metabolome of infected macrophages beyond an increase in glycolysis, the untargeted metabolic profiling often enables only tentative structural annotation. Therefore, to complement our untargeted dataset, we employed ^1^H-nuclear magnetic resonance (NMR) spectroscopy to further dissect the metabolic effects of *Mtb* infection in M2 macrophages compared to uninfected control samples at 4 h and 24 h post-infection. While not as sensitive as mass spectrometry, ^1^H-NMR spectroscopy is nonetheless quantitative and known for its robustness^34^.

**Figure 4:**
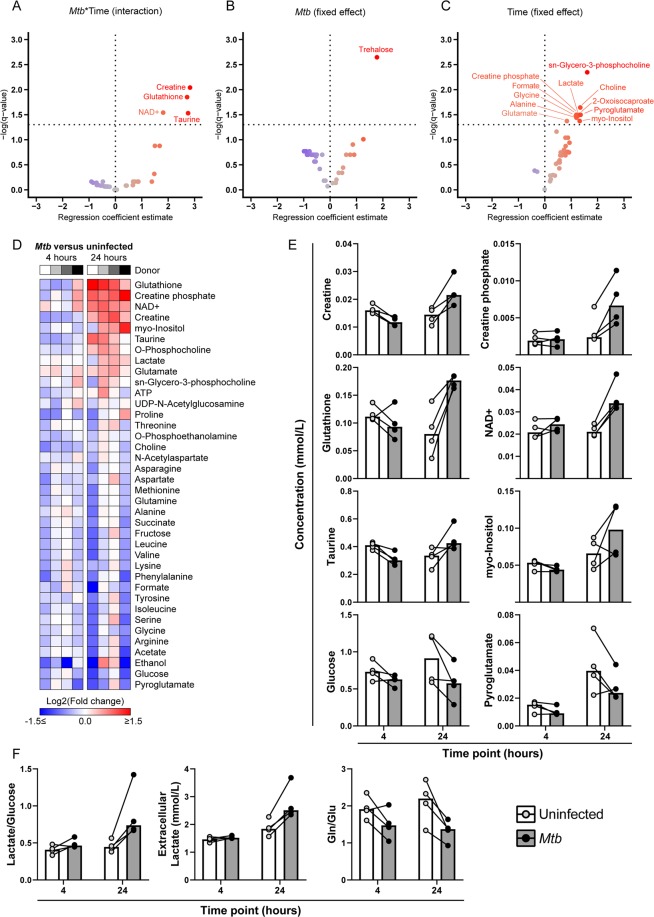
Targeted ^1^H-NMR spectroscopy analysis of *Mtb*-infected M2 macrophages. M2 macrophages were infected with *Mtb* at a MOI of 10:1 or left uninfected. Cells were lysed at 4 h and 24 h post-infection and intracellular metabolites were subsequently extracted and measured by ^1^H-NMR spectroscopy. Linear random intercept models for fitted for each metabolite to investigate: (**A**) the interaction between infection and time (*Mtb*:Time), or the separate fixed effects of (**B**) *Mtb* infection or (**C**) time. Resulting -log-transformed FDR-corrected *p*-values (*q*-values) are plotted against the regression coefficient estimate for each metabolite in volcano plots. (**D**) Heatmap of log_2_-transformed metabolite fold changes (*Mtb*/uninfected). Metabolites are sorted by average median fold change at 24 h. Individual donors are presented in greyscale. (**E**) Absolute levels of creatine, creatine phosphate, glutathione, NAD^+^, taurine, myo-inositol, glucose and pyroglutamate (mmol/L) in *Mtb*-infected macrophages (grey bars) or uninfected controls (white bars) at 4 h and 24 h. (**F**) Extracellular lactate concentrations and relative ratios of lactate/glucose and glutamate/glutamine (Glu/Gln). Each dot represents an individual donor, and measurements from matching donors are connected by black lines. Bars represent group medians.

Separate linear random intercept models were fitted for each individual metabolite to model the interaction between infection status (*Mtb* vs uninfected) and time (24 h vs 4 h) (Fig. 4A). Significant interactions were detected for four metabolites, creatine (*q* = 9.09E^−3^), glutathione (*q* = 0.014), nicotinamide-adenine-dinucleotide (NAD^+^) (*q* = 0.029) and taurine (*q* = 0.029), all of which increased between 4 and 24 h in infected macrophages but either decreased or did not change in uninfected controls (Fig. 4E). Together with creatine-phosphate and myo-inositol, these metabolites constituted the top six most elevated factors in *Mtb*-infected versus uninfected macrophages according to median log_2_-transformed fold changes (Fig. 4D,E). Next, we fitted linear mixed models without interaction term to analyze the fixed effects of infection and time. The disaccharide trehalose, an important component of mycobacterial cell-wall glycolipids^35^, was only detected in infected macrophages and therefore significantly associated with *Mtb* infection (*q* = 2.26E^−3^) (Fig. 4B). Various metabolites showed a significant positive association with time (Fig. 4C), including sn-glycero-3-phosphocholine (*q* = 4.50E^−3^), choline (*q* = 0.023), creatine phosphate (*q* = 0.032), pyroglutamate (*q* = 0.032) and lactate (*q* = 0.033), although the magnitude of this increase could vary based on infection status (Fig. 4E).

As macrophage activation with *Mtb* lysate induced a relative increase in extracellular acidification indicative of anaerobic glycolysis, we wondered whether live *Mtb* infection would also trigger similar changes in glycolytic intermediates. While glucose levels were lower in *Mtb*-infected macrophages in 3/4 donors at both 4 h and 24 h, the lactate/glucose ratio was increased in all donors at 24 h (median increase of 81%) (Fig. 4F), indicative of increased glucose utilization for ATP production by anaerobic glycolysis^36^, which is congruent with the results from Fig. 1. Additionally, extracellular lactate concentrations were elevated in all donors at 24 h post-infection (Fig. 4F). As only one intermediate of the TCA cycle was detected (succinate) which in itself did not show obvious modulation by *Mtb* infection, the relative mitochondrial activity could not be assessed in a similar fashion. However, we did observe a decreased glutamine (Gln) to glutamate (Glu) ratio (Fig. 4F), reflecting increased glutamine catabolism.

### *Mtb*-induced metabolic changes are reflected by the macrophage transcriptome

**Figure 5:**
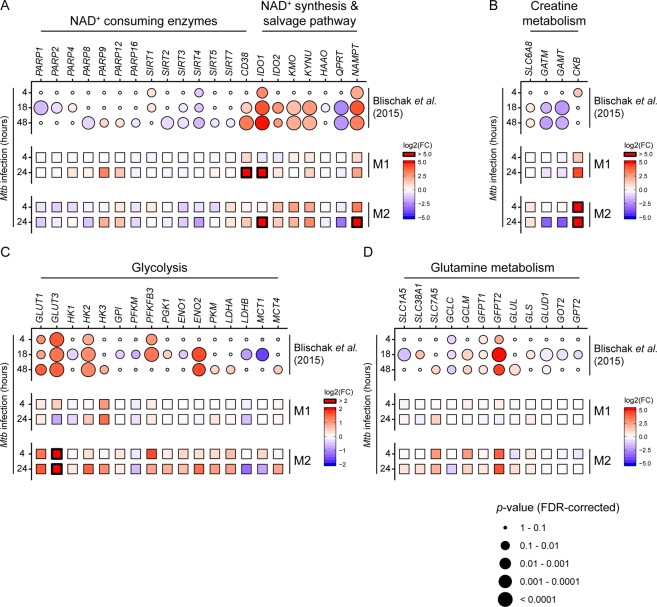
*Mtb* modulates gene expression of key metabolic enzymes in infected macrophages. Expression profiles of genes involved in (**A**) NAD^+^ consumption and synthesis, (**B**) creatine metabolism, (**C**) glycolysis and (**D**) glutamine metabolism from a previously published RNA-seq dataset (Blischak *et al*.)^37^ of M2 macrophages at 4, 18 and 48 h post-*Mtb* infection and an exploratory RNA-seq dataset derived from our M1/M2 *Mtb* infection model. Expression data is displayed as log_2_-transformed fold changes of *Mtb* infected vs non-infected macrophages. Up- and downregulation of genes is displayed by a color gradient (up = red, down = blue). Differential expression results (FDR-corrected *p*-values) from Blischak *et al*. are reflected by circle size.

Finally, we wondered whether the observed metabolic changes induced by *Mtb* infection were reflected by alterations in macrophage gene expression levels. To study this, we analysed the results of previously published expression profiling of M2 macrophages infected with *Mtb*-H37Rv (n = 6) performed by Blischak *et al*.^37^ for differentially expressed genes involved in glycolysis, NAD^+^, creatine and glutamine metabolism, and compared their expression profiles to an exploratory RNA-seq dataset of *Mtb*-infected M1 and M2 macrophages acquired using our own specific *Mtb* infection model. Gene expression profiles showed significant linear correlation between both datasets, especially for M2 macrophages at 24 h (Fig. S5). In both sets, *Mtb* infection was associated with differential expression of NAD^+^-consuming enzymes, such as cyclic ADP ribose hydrolase (*CD38*) and various members of the poly (ADP-ribose) polymerase (PARP) and sirtuin (SIRT) protein families (Fig. 5A). Congruent with the observed increase in intracellular NAD^+^ levels, genes involved in NAD^+^ biosynthesis were strongly upregulated during *Mtb* infection, including nicotinamide phosphoribosyltransferase (*NAMPT*) and indoleamine 2,3-dioxygenase 1 (*IDO1*), rate-limiting enzymes of the NAD^+^ salvage and kynurenine pathway respectively. In contrast, expression of quinolate phosphoribosyltransferase (*QPRT*), which catalyzes quinolinic acid conversion downstream of *IDO1*, was decreased. With regard to creatine metabolism, expression of creatine synthesis enzyme genes *GATM* and *GAMT* decreased as a result of *Mtb* infection, while the creatine transporter *SLC6A8* and creatine kinase (brain-type, *CKB*) were increased, most notably in our M2 infection model (Fig. 5B). Furthermore, *Mtb* infection induced expression of genes known to control glycolytic flux^38^, namely glucose transporter 1 (*GLUT1*) and 3 (*GLUT3*), hexokinase 2 (*HK2*), 6-phosphofructo-2-kinase/fructose-2,6-biphosphatase 3 (*PFKFB3*) and monocarboxylate transporter 4 (*MCT4*) (Fig. 5C), which is again in agreement with the NMR metabolic data. Finally, *Mtb* modulated expression of various genes involved in glutamine metabolism in both datasets, including members of the hexosamine (*GFPT1* and *GPFT2*) and glutathione synthesis (*GCLC* and *GCLM*) pathways (Fig. 5D), corresponding with the increased levels of intracellular glutathione after *Mtb* infection. Taken together, we find that *Mtb* infection results in clear metabolic changes in macrophages, including alterations in NAD^+^, creatine, glucose and glutamine metabolism, which can be connected to corresponding changes in metabolic gene expression patterns.

## Discussion

Since the emergence of the multidisciplinary field of immunometabolism, a growing body of evidence has accumulated connecting specific aspects of cellular metabolism to macrophage activation and function in response to danger signals or microbes. While we were able to validate that activation with LPS and *Mtb* lysate resulted in a relative shift toward increased glycolysis in primary macrophages, many of the published findings in the literature have yet to be translated to infections of primary human cells with live pathogens. Here, we contribute to this knowledge gap by employing both untargeted LC-MS metabolomics and targeted ^1^H-NMR spectroscopy to investigate the effect of live *Mtb* infection on macrophage metabolism. PLS-DA modeling of untargeted metabolomics data demonstrated that both *Mtb* infection and LPS stimulation were associated with marked changes in the cellular metabolome in both M1 and M2 macrophages. Tentative annotation of changed metabolites indicate increased levels of adenosine, nicotinamide and propionylcarnitine in LPS-stimulated M1 and M2 macrophages. *Mtb* infection was strongly associated with increased levels of relatively large masses, however these could not be annotated based on current metabolite databases and potentially constitute *Mtb*-derived protein fragments or lipids, one of which was identified as trehalose using quantitative ^1^H-NMR measurements. The latter also revealed various changes in specific metabolites in *Mtb*-infected versus uninfected macrophages, including NAD^+^, creatine and glutathione, which could be linked to changes in gene expression as determined by analysis of RNA-seq datasets.

Levels of NAD^+^, an important cofactor in many cellular pathways, were elevated in *Mtb*-infected macrophages. *Mtb* has been demonstrated to secrete toxins which can induce macrophage necrosis through NAD^+^-depletion^39,40^. In addition, recent studies have highlighted the importance of sustaining adequate NAD^+^ levels for pro-inflammatory macrophage function. Reactive oxygen species (ROS) produced during inflammatory activation were shown to induce DNA damage in macrophages, leading to PARP activation and depletion of NAD^+^ pools^41^. NAD^+^ salvage through activation of NAMPT was required for maintaining glycolytic flux and inflammatory functions. Furthermore, NAD^+^ synthetic capacity was reduced in aged mice compared to young controls, indicating that changes in NAD^+^ metabolism could play a role during aging-associated immune dysregulation^42^. Our results imply increased activity of NAD^+^ biosynthesis pathways as a result of *Mtb* infection. This result is corroborated by analysis of independent RNA-seq datasets, which showed increased expression of genes involved in NAD^+^ synthesis and salvage, including IDO1 and NAMPT. Interestingly, expression of QPRT was decreased in M2 macrophages, an effect which was found to limit *de novo* NAD^+^ synthesis through the kynurenine pathway in LPS-stimulated macrophages^42^. As the general interest in the therapeutic potential of NAD^+^-boosting drugs is on the rise^43^, these results call for further studies on the importance of macrophage NAD^+^ metabolism during *Mtb* infection, also in view of potential application in host-directed therapeutic strategies.

Besides NAD^+^, intracellular levels of the anti-oxidant glutathione (GSH; reduced form) also increased during macrophage *Mtb* infection. Glutathione is produced as part of the cellular redox response to increased levels of ROS and regulates macrophage anti-mycobacterial functions both through direct anti-microbial effects and by serving as a carrier of nitric oxide (NO)^44^. Furthermore, *Mtb*-infected cells showed an increase in glutamine catabolism via its conversion to glutamate. The latter is a precursor for the *de novo* synthesis of glutathione and together with the increased intracellular GSH, point to a metabolic switch caused by *Mtb* infection towards the synthesis of glutathione^45^. As glutamine has been reported to regulate macrophage cytokine production^46^ and support M2 polarization^47^, increased glutamine catabolism could potentially modulate macrophage cytokine responses to *Mtb*, although the resulting net effect on mycobacterial bacterial survival is unclear. Intracellular levels of creatine and creatine phosphate were also increased after 24 h of *Mtb* infection. The creatine phosphate system constitutes a spatiotemporal buffer for intracellular ATP concentrations, mostly in tissues with high energy consumption such as skeletal muscle and brain^48^. Creatine phosphate was also shown to be involved in cytoskeletal dynamics^49,50^ and macrophage phagocytosis^51^, possibly through increased localization of CKB to nascent phagosomes^52^. The observed increase in creatine phosphate and CKB expression could therefore be a reflection of macrophage phagocytic activity. Additionally, creatine is synthesized from arginine, and increased creatine production could reduce intracellular availability of arginine for anti-bacterial NO production^53^. However, as creatine synthesis *in vivo* occurs through respective actions of GATM of GAMT in the kidneys and liver, the elevated creatine levels could also be the result of increased expression of the creatine transporter SLC6A8, as was observed in the RNA-seq analysis. Finally, the intracellular lactate/glucose ratio was elevated during *Mtb* infection, further evidencing an increased glycolytic flux from glucose to lactate. These findings are corroborated by a recent study which also reported increased levels of lactate and glutamine-derived metabolites in *Mtb* lysate-stimulated peripheral blood mononuclear cells^54^. Interestingly, while increased glycolysis has been linked to improved *Mtb* clearance^21^, a recent paper demonstrated that *Mtb* can utilize lactate as a source of carbon for intracellular replication in macrophages^55^, calling for more extensive studies on the role of lactate production during macrophage *Mtb* infection.

Our study has several limitations that need to be discussed. Firstly, it was not possible to differentiate whether metabolites were of mycobacterial or human host origin in our experimental setup, with the exception of metabolites such as trehalose that can only be of mycobacterial origin, and were only detected in *Mtb* infected macrophages. However, we consider major mycobacterial contributions to metabolite levels unlikely due to the large difference in cell volume between macrophages and infecting *Mtb*. Furthermore, the majority of our observed changes (e.g. elevated creatine, glutathione, NAD^+^) manifested predominantly with prolonged infection time, which could be considered an argument against being mycobacterial metabolites as these were also present and likely measurable at 4 h, in a similar fashion to trehalose. Secondly, we were unable to perform extracellular flux analysis during live *Mtb* infection due to biosafety restrictions and therefore used *Mtb* lysate as a model for infection. While the observed increase in ECAR/OCR ratio is corroborated by other studies using γ-irradiated *Mtb*^21,56^, this result was contradicted by a recent paper which reported an overall decrease in macrophage bioenergetic profile during live *Mtb* infection^57^. Although we find that a potential role of toxicity on these results is not conclusively excluded by the authors, it would still be important to compare the effects of *Mtb* lysate stimulation to those of live *Mtb* infection in future experiments. Lastly, it is unclear how accurately the observed metabolic responses of human monocyte-derived macrophages reflect those of alveolar macrophages *in vivo*. Results from mouse studies are suggestive of a model in which the metabolic response of macrophages to *Mtb* infection is largely dependent on their ontology^58^. Therefore, it would be of great interest to study whether our results are reproducible using primary tissue-resident macrophage populations.

In conclusion, live *Mtb* infection induces pronounced metabolic changes in primary human macrophages, including activation of NAD^+^ and glutathione synthesis, glycolysis, glutaminolysis and the creatine phosphate pathway. Whether these changes benefit the host or the bacterium could not directly be inferred from these experiments and needs to be studied further: follow-up experiments using small-molecule inhibitors or small interfering RNAs (siRNAs) which target key enzymes of these pathways will be necessary to gauge their exact involvement in the macrophage anti-mycobacterial immune response.

## Materials and Methods

### Study design and sample collection

All experimental procedures were performed according to local and national guidelines on the work with pathogenic mycobacteria. Infection experiments with *Mycobacterium tuberculosis H37Rv* were performed as approved by the local biosafety officer and following the BSLIII permit granted to LUMC by the Dutch government.

Monocytes were isolated from buffy coats obtained from Sanquin blood products (Amsterdam, The Netherlands). The medical ethical review board of LUMC has approved use of buffy coats, remaining after blood donation, for scientific purposes. Healthy donors donating their blood have provided written informed consent for scientific use of their blood products.

### Monocyte isolation and differentiation

CD14^+^ monocytes were isolated from buffy coats of healthy blood bank donors by positive selection using an autoMACS Pro Separator (Miltenyi Biotec BV, Leiden, The Netherlands). Monocytes were differentiated into macrophages using 50 ng/ml M-CSF (Miltenyi Biotec) or 5 ng/ml GM-CSF (Miltenyi Biotec) for six days at 37 °C/5%CO_2_. Cells were cultured in RPMI-1640 medium supplemented with 10% fetal calf serum (FCS), 100 units/ml penicillin and 100 µg/ml streptomycin and GlutaMAX (Gibco, Thermo Fisher, Merelbeke, Belgium). After differentiation macrophages were harvested by trypsinization and seeded in multiwell plates. As a quality control, macrophages were stained for surface expression of CD14 and CD163 and acquired on a BD LSRFortessa flow cytometer (BD Biosciences, Erembodegem, Belgium).

### Seahorse extracellular flux (XF) analysis

For cellular metabolic flux analysis, macrophages were stimulated overnight with either medium, 100 ng/ml LPS or H37Rv *Mtb* lysate (10 µg/ml) and measured on a Seahorse XF96 Analyzer (Seahorse Bioscience, North Billerica, MA, USA). Cell culture medium was replaced with RPMI without buffer and glucose supplemented with 5% FCS and L-glutamine and macrophages were incubated in a 37 °C dry incubator for one hour before start of measurements. Macrophage oxygen consumption rates (OCR) and extracellular acidification rates (ECAR) were determined in real-time throughout consecutive injections of D-glucose (10 mM), oligomycin (1 µM) and FCCP (2 µM). Acidification due to glycolysis was calculated as the difference in highest ECAR measurements pre- and post-glucose injection. Oxygen consumed for ATP production by oxidative phosphorylation was calculated as the difference between highest OCR measurements pre- and post-oligomycin injection. In case of obvious injection errors the affected measurements were excluded from the analysis.

### *Mtb* H37Rv culture and infection

*Mtb* H37Rv cultures were grown to mid-log phase in Middlebrook 7H9 liquid medium (Difco, BD Biosciences) supplemented with albumin/dextrose/catalase (ADC) (BBL, BD Biosciences). Bacterial concentrations were determined by measuring the culture optical density at 600 nm^59^. Macrophages were infected with H37Rv at a multiplicity of infection (MOI) of 10:1 for 1 hour at 37 °C, after which the cells were washed twice with medium containing 30 µg/ml gentamicin and further cultured for 4 or 24 h in fresh medium containing 5 µg/ml gentamicin. MOI was confirmed by plating a dilution series of the inoculum on 7H10 square agar plates supplemented with oleate/albumin/dextrose/catalase (OADC) (BBL, BD Biosciences)^59^.

### Sample preparation for LC-MS

Macrophages were seeded in 24-well plates at a density of 300,000 cells/well and either infected with *Mtb*, stimulated with LPS (100 ng/ml; Thermo Fisher) or left untreated. At 4 and 24 h post-infection/stimulation, macrophages were washed with ice-cold 1% NaCl and subsequently lysed in water by osmotic pressure for 15 minutes at 4 °C. Lysates were thoroughly resuspended and mixed with pre-heated 80% ethanol at a 1:3 ratio (end concentration: 60% ethanol) in polypropylene screwcap tubes and subsequently heated for 10 min at 90 °C. Samples were chilled for 10 minutes on ice before centrifugation at 13.2 × 1000 rpm for 10 minutes at 4 °C, after which the supernatants were harvested and stored at −80 °C for subsequent LC-MS analysis.

### LC-MS/MS measurements and metabolite annotation

Samples were randomized before the analysis. The acquisition sequence was designed using a standard block structure: the material was injected by the blocks of five samples flanked by the QC pool samples. Fifty µL of each sample or QC pool were injected into the RPLCQ-TOF system (Ultimate 3000RS tandem Ultra High Performance Chromatography system, Thermo Scientific/Dionex, Amsterdam, Netherlands; Electrospray Ionization – Ultra High Resolution - Time of Flight mass spectromter maXis, Bruker Daltonics, Bremen, Germany). The details of the RPLC-Q-TOF method have previously been reported^60^. Pre-processing of the raw data (alignment of retention time, peak picking, filtering and normalization) was performed on the files converted into mzxml format. Retention time was aligned using the msalign package^61^ keeping the mass error parameter at 5 ppm. Peak picking and grouping were done within the XCMS package using centWave function^62^. A final data matrix included only the features with a relative standard deviation ≤0.3 within the QC pool. Finally, we manually removed the signals corresponding to the HEPES clusters and anticipating the potential difficulties with structural annotation, which are known for the of the untargeted profiling data, we filtered out the last quarter of the chromatogram.

Metabolite annotation was carried out according to the minimal reporting standards^63^. The Smart Formula tool within the Data Analysis software (version 4.1) as used for the initial ion annotation based on accurate mass (mass error < 5 ppm) and isotopic distribution (sigma value < 20). The results were matched against online metabolomics databases (METLIN, Human Metabolome Database, MassBank). When possible the hits were confirmed with MS–MS experiments of the sample with the highest intensity for each of the putative metabolites and the reference standards. MS–MS experiments were performed on the same RPLC-Q-TOF instrument in auto MS–MS mode.

### Sample preparation for ^1^H-NMR spectroscopy

Macrophages were seeded in 24-well plates with 300,000 cells/well and either infected with H37Rv or left untreated. At 4 and 24 h post-infection, supernatants were harvested and the cells were washed once quickly with ice-cold PBS. Macrophages were rapidly quenched with liquid nitrogen and the plates were stored at −80 °C. Supernatants were filter-sterilized using 2 µM filter plates, mixed with methanol chilled at −80 °C at a ratio of 1:3 and subsequently stored at −80 °C. On the day of metabolite extraction, plates were put on ice and 300 µl 90% methanol/chloroform 9:1 was added to each well and cells were scraped thoroughly with a pipette tip before transferring of samples to eppendorf tubes. Samples were chilled for 10 minutes on ice before centrifugation at 13.2 × 1000 rpm for 15 minutes at 4 °C, after which the supernatants were harvested and put on ice. Dry protein pellets were stored at −20 °C and protein concentrations were determined by bicinchoninic acid assay (BCA) (Pierce, Thermo Fisher) according to manufacturer’s instructions. Supernatant/methanol samples from the −80 °C were centrifuged at 13.2 × 1000 rpm for 30 minutes at 4 °C, after which supernatants were collected and also put on ice. Both cellular- and supernatant- extracts were then dried by nitrogen stream and stored at −80 °C until day of measurement.

### ^1^H-NMR spectroscopy

NMR analysis of the intracellular metabolites was carried out as described previously^34^. Briefly, the dried extracts were reconstituted in 250 µl of 0.15 M K_2_HPO_4_/KH_2_PO_4_ buffer (pH = 7.4) in 99.9% deuterated water (D_2_O), including 0.2 mM NaN_3_ and 0.4 mM trimethylsilylpropionic acid sodium salt (TSP-*d*_4_), and transferred to 3-mm NMR tubes. NMR data were recorded on a 14.1 T NMR spectrometer (600 MHz for ^1^H; Bruker Avance II) under standardized conditions for all samples. All spectra were processed for phase and baseline correction and referenced to TSP-*d*_4_. One-dimensional (1D) spectra were imported into Chenomx NMR suit 8 (Chenomx Edmonton, Canada) for quantification. Metabolites were identified based on the Bbiorefcode (Bruker Biospin) and the Chenomx databases as well as in-house reference spectra. The concentrations of the quantified metabolites (mM) were normalized to the protein mass per sample.

### RNA-sequencing

Total RNA was extracted from of M1 and M2 macrophages either uninfected or infected with *Mtb* in triplicate from a single blood bank donor using TRIzol Reagent (Thermo Fisher) at 4 and 24 h post-infection and purified using RNeasy MinElute Cleanup Kit (Qiagen, The Netherlands). The concentration and purity of RNA was evaluated by NanoDrop 2000 (Thermo Fisher). RNA-seq was performed using an Illumina Hi-Seq. 2500 as previously described^64^. The RNA-seq data were mapped versus the human genome (version GRCH38) and tag counts were performed by Bowtie 2 using GeneTiles software (http://www.genetiles.com)^65^. Normalization and gene expression analysis was performed using the R package DESeq2^66^. The RNA-seq dataset has been deposited in NCBI’s Gene Expression Omnibus (GEO) and are accessible through GEO Series accession number GSE148731. https://www.ncbi.nlm.nih.gov/geo/query/acc.cgi?acc=GSE148731.

### Statistical analysis

All statistical methods were performed in R (version 3.5.0) or GraphPad software (version 7.02, Prism, La Jolla, CA, USA). Morpheus (https://software.broadinstitute.org/morpheus) was used to generate the NMR metabolite heatmap. The following R packages were used: (multilevel) PCA and PLS-DA modeling was performed using mixOmics version 6.3.2^67^, linear mixed models were fitted using lme4 version 1.1.17^68^ and lmerTest^69^ version 3.0.1, and graphical output was constructed using ggplot2 version 3.1.0^70^.

## Supplementary information

Supplementary information.

Supplementary information 2.

Supplementary information 3.

Supplementary information 4.

Supplementary information 5.

Supplementary information 6.
