# Supplementary figures and images for: Analyzing the impact of Mycobacterium tuberculosis infection on primary human macrophages by combined exploratory and targeted metabolomics
Source: Sci Rep. 2020 Apr 27;10:7085. doi: 10.1038/s41598-020-62911-1 (PMC7184630; doi:10.1038/s41598-020-62911-1)

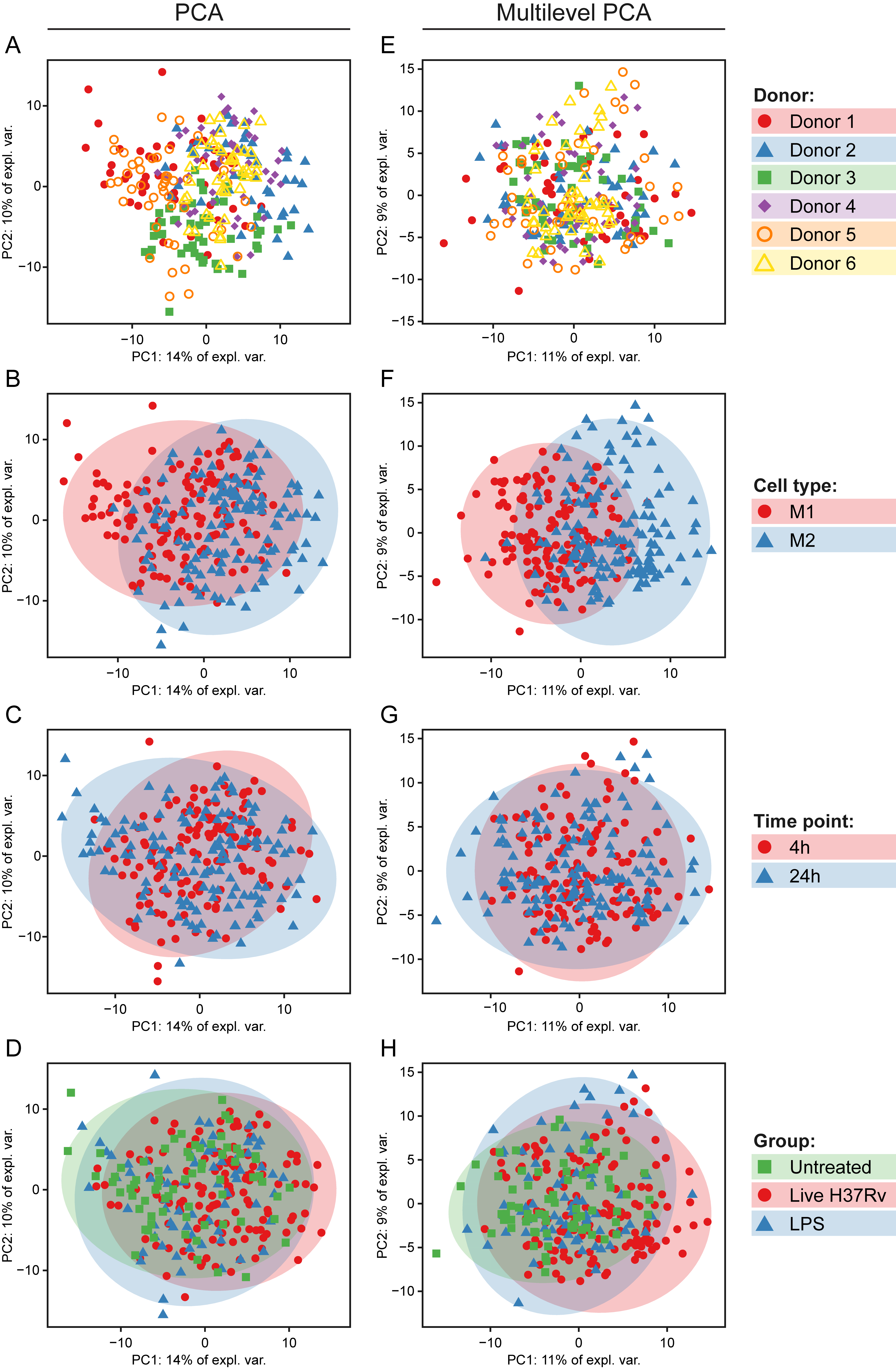

Supplement: Supplementary file 1 — Supplementary information. [file 41598_2020_62911_MOESM1_ESM.tif]

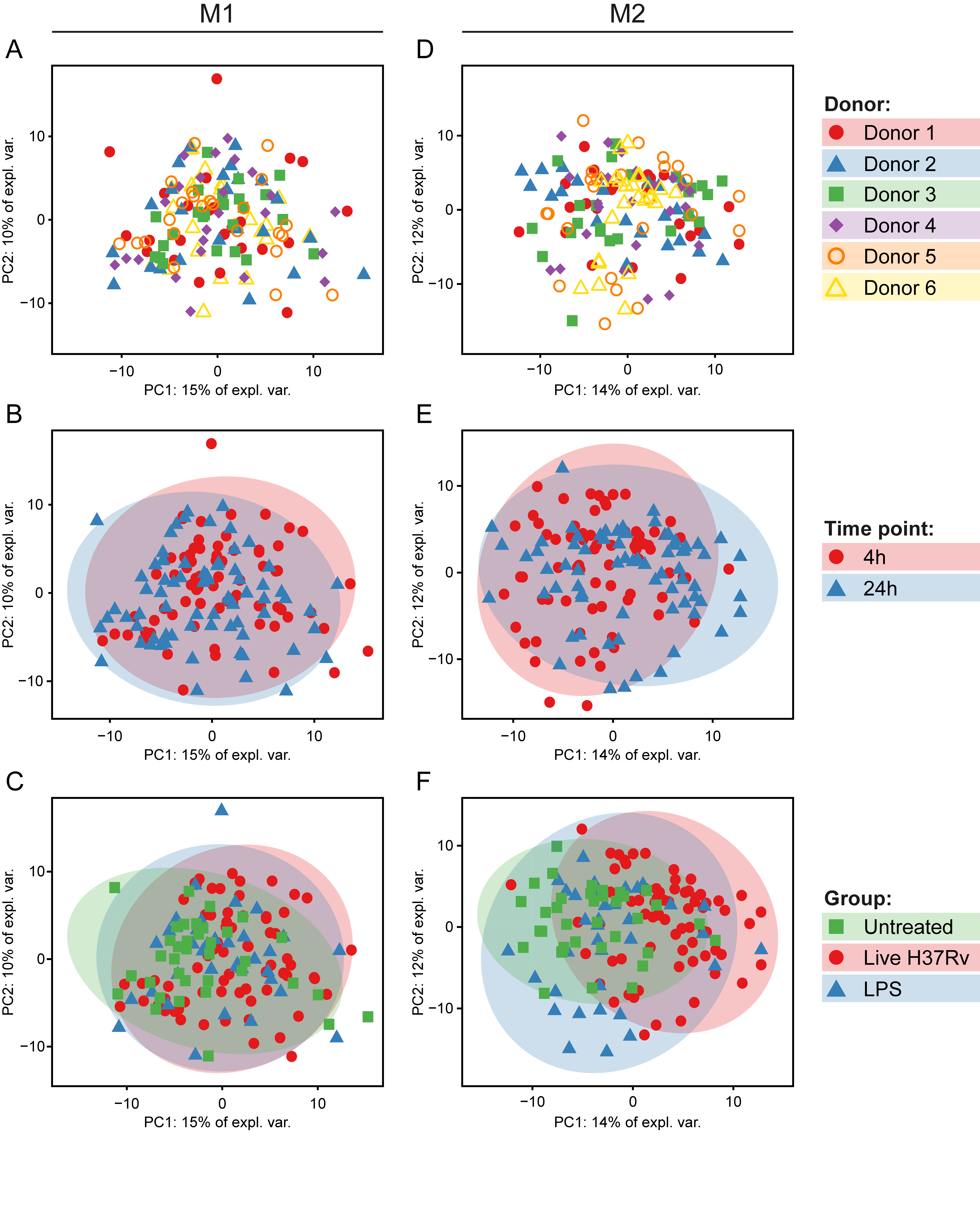

Supplement: Supplementary file 2 — Supplementary information 2. [file 41598_2020_62911_MOESM2_ESM.tif]

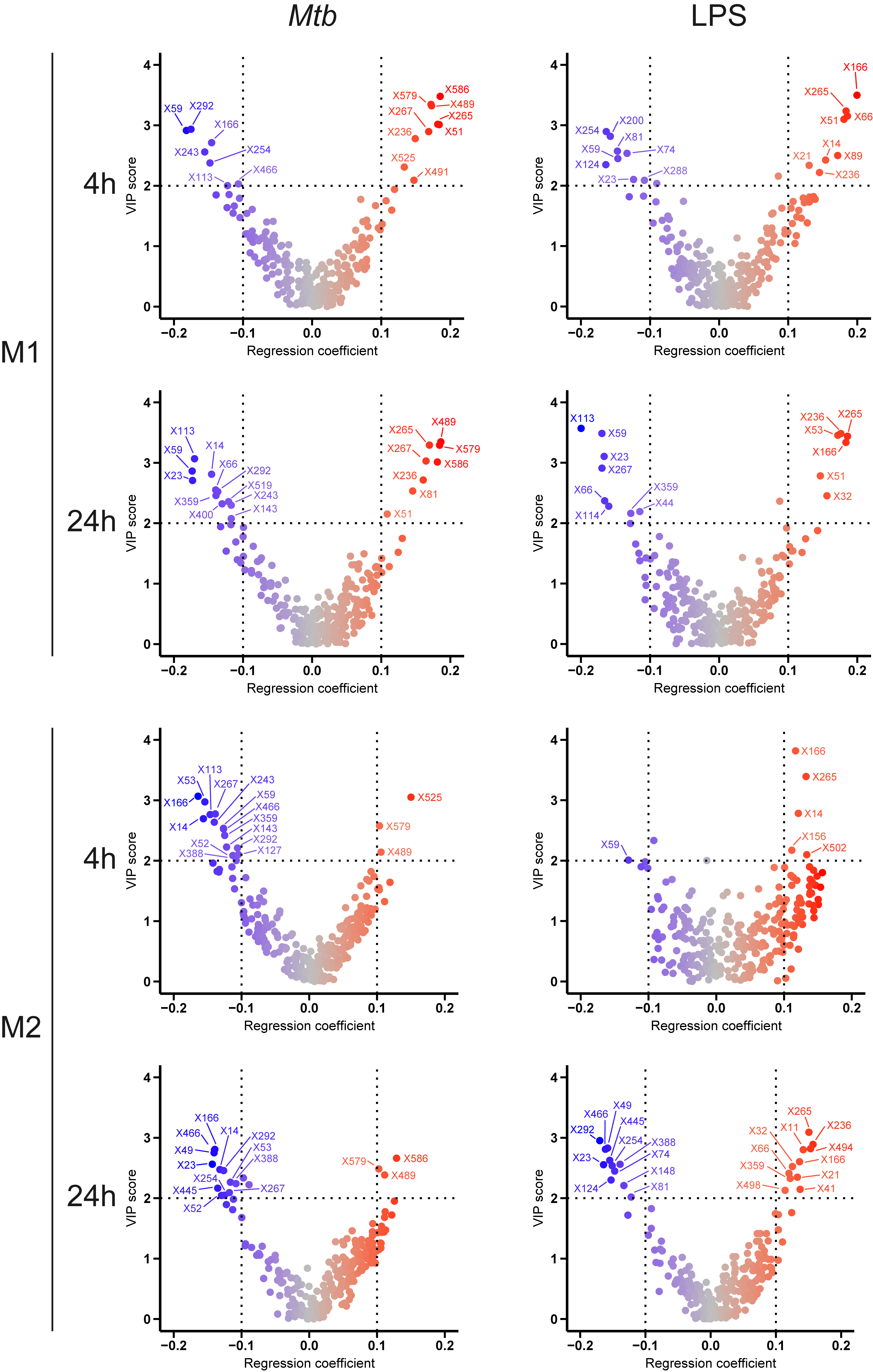

Supplement: Supplementary file 3 — Supplementary information 3. [file 41598_2020_62911_MOESM3_ESM.tif]

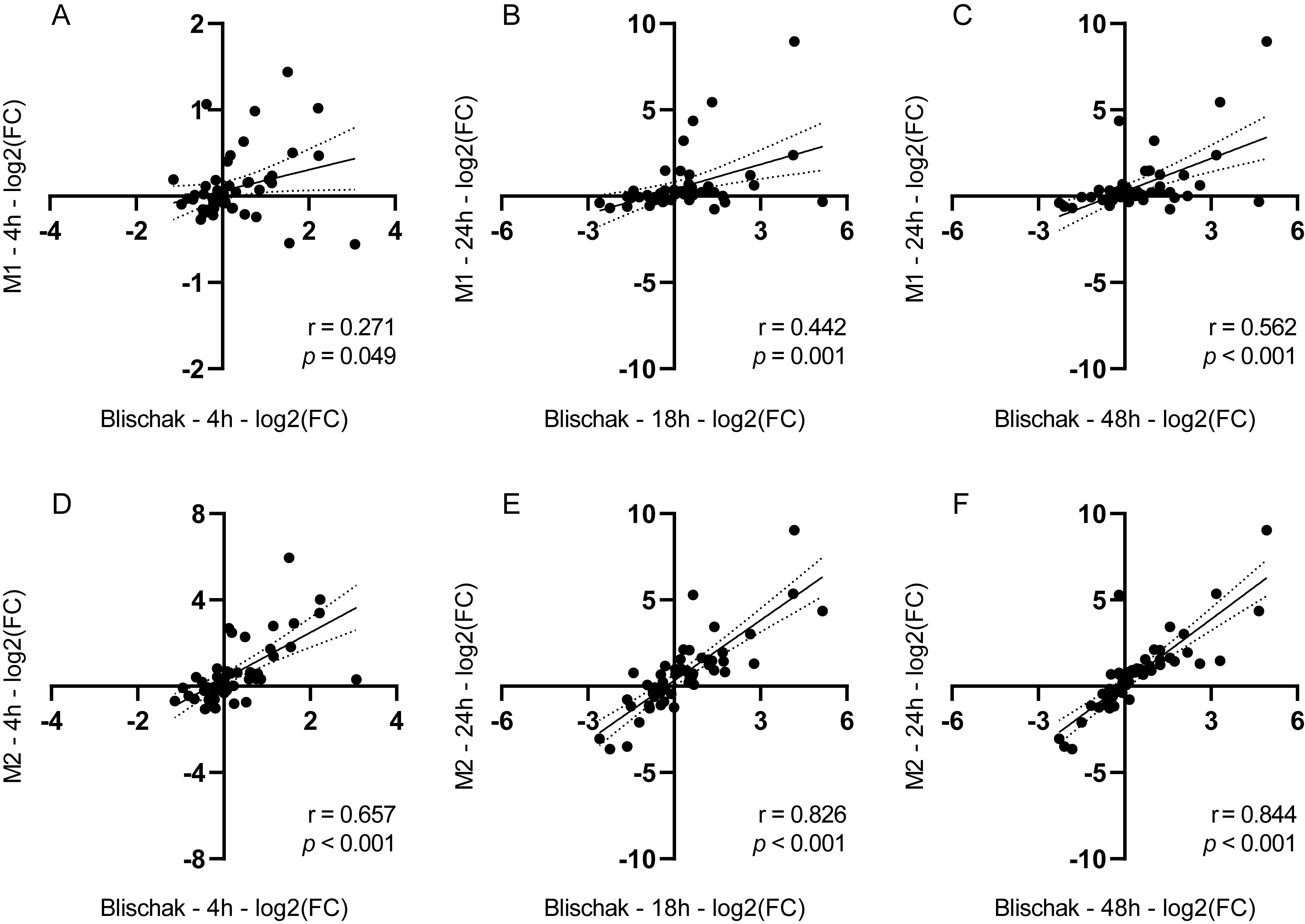

Supplement: Supplementary file 5 — Supplementary information 5. [file 41598_2020_62911_MOESM5_ESM.tif]
