# Supplementary material for: Analyzing the impact of Mycobacterium tuberculosis infection on primary human macrophages by combined exploratory and targeted metabolomics
Source: Sci Rep. 2020 Apr 27;10:7085. doi: 10.1038/s41598-020-62911-1 (PMC7184630; doi:10.1038/s41598-020-62911-1)
Supplement: Supplementary file 6 — Supplementary information 6. [file 41598_2020_62911_MOESM6_ESM.docx]

**Figure S1:** **PCA models of untargeted LC-MS metabolomics.** Score plots of the first two components of a standard (A-D) and a multilevel PCA (E-H) model built on the entire dataset. Samples are color coded by donor (A&E), cell type (B&F), time point (C&G) or group membership (D&H).

**Figure S2:** **Separate multilevel PCA models by cell type.** Score plots of the first two components of multilevel PCA models built for M1 (A-C) or M2 macrophages (D-F). Samples are color coded by donor (A&D), time point (B&E) or group membership (C&F).

**Figure S3:** **PLS-DA model volcano plots.** VIP scores and associated regression coefficients were extracted from *Mtb*- and LPS-derived multilevel PLS-DA models for each metabolite at 4 h and 24 h and displayed as volcano plots. VIP score and regression coefficient cut-offs levels of 2 and -0.1/0.1 are shown as horizontal and vertical dashed lines respectively.

**Figure S4: Abundance of selected metabolites in M1 and M2 macrophages.** Peak area results of 21 masses with high correlation scores (≥ 0.5) in the final M1 and M2 PLS-DA models. Results are displayed in Tukey’s boxplots for untreated (white), *Mtb*-infected (light gray) and LPS-stimulated (dark gray) M1 or M2 macrophages at 4 and 24 h.

**Figure S5: Metabolic gene expression profiles of Blischak *et al.* and exploratory M1/M2 RNA-seq data showed high positive correlation.** Comparison of macrophage gene expression profiles of 53 metabolic genes during *Mtb* infection between Blischak *et al.* (37) and the exploratory M1/M2 RNA-seq dataset. Correlation between: (A) M1 macrophages (4 h) versus Blischak *et al.* (4 h), (B) M1 macrophages (24 h) versus Blischak *et al.* (18 h), (C) M1 macrophages (24 h) versus Blischak *et al.* (48 h), (D) M2 macrophages (4 h) versus Blischak *et al.* (4 h), (E) M2 macrophages (24 h) versus Blischak *et al.* (18 h) and (F) M2 macrophages (24 h) versus Blischak *et al.* (48 h). Expression data is plotted as log_2_-transformed fold changes of *Mtb* infected vs non-infected macrophages with linear regression line and 95% confidence interval. Individual genes are depicted as dots. Pearson correlation coefficient (r) and associated *p*-value are displayed for each comparison.
